# Welding Fumes in a Chinese Shipyard: Exposure Characteristics and Occupational Health Risk Assessment

**DOI:** 10.3390/toxics14030259

**Published:** 2026-03-16

**Authors:** Yulu Hu, Jingbo Zhang, Xiangpei Lyu, Chunhui Ni, Huanqiang Wang

**Affiliations:** 1 National Institute of Occupational Health and Poison Control, Chinese Center for Disease Control and Prevention, Beijing 100050, China; huyulu2001@163.com (Y.H.); lvxp@niohp.chinacdc.cn (X.L.); 2 Department of Toxicology, Shanghai Pulmonary Hospital, Tongji University, Shanghai 200433, China; 13585506860@163.com; 3 School of Public Health, Nanjing Medical University, Nanjing 211166, China

**Keywords:** shipyards, welding fumes, mass concentration, count concentration, particle size distribution, metal exposure, risk assessment, confined space

## Abstract

Welding fumes in the shipbuilding industry severely threaten workers’ health. This study systematically investigated welding fume exposure in a Chinese shipyard, analyzing mass concentration, particle size distribution, and harmful metal content using data from 2015. Differences were observed across welding sites and processes. Confined spaces and gas metal arc welding (GMAW) were associated with significantly higher exposure levels. Welding fumes were dominated by particles smaller than 1.00 μm, a distribution influenced by welding site, distance from the welding spot, and process. Iron (Fe) and manganese (Mn) were the predominant metal components, with concentrations significantly higher in respirable dust than in total dust. Risk assessment indicated minimal non-cancer hazards for Fe, zinc, and copper. However, Mn posed the predominant risk (Hazard Quotient >> 1), while nickel (Ni) and chromium (Cr) also exceeded safety thresholds at most points. Consequently, confined spaces and GMAW should be prioritized as key control targets in shipyards, as respirable dust rich in metal-bearing particles poses greater health risks. Therefore, China urgently requires the establishment of specific occupational exposure limits for respirable welding fumes. Additionally, personal sampling is more focused and efficient than area sampling for precise occupational health risk assessment due to the greater mobility of welding operations.

## 1. Introduction

Welding is an essential industrial process widely applied in critical sectors, including shipbuilding, petrochemicals, construction, automotive manufacturing, aerospace, and defense. Welding fume, generated at high temperatures (2000–6000 °C) via the melting, evaporation, and oxidation of welding consumables and base metals, is the primary occupational hazard in welding operations [1]. Approximately 94.2% of the welding fume particles are smaller than 5 μm, typically ranging from 0.01 to 0.4 μm, with high dispersibility [2] (pp. 122–123). Due to their fine size, the majority of welding fumes can be directly inhaled into the deep lungs, posing significant health risks. Extensive evidence has linked welding fume exposure to adverse health outcomes. Welding fumes pose a significant threat to respiratory health, inducing disorders such as pulmonary dysfunction [3], asthma [1], bronchitis [4], and chronic obstructive pulmonary disease (COPD) [5], and also markedly increasing the risk of malignant tumors, including lung cancer [6]. Furthermore, they can trigger cardiovascular damage and neurological impairment [7,8]. The International Agency for Research on Cancer (IARC) classified welding fumes as carcinogenic to humans (Group 1) in 2017 [9]. The global burden of occupational exposure to particulate matter, gases, and fumes is substantial. According to the Global Burden of Disease Study 2021, occupational exposure ranked as the fourth leading risk factor for chronic respiratory diseases worldwide [10].

The National Institute for Occupational Safety and Health (NIOSH) reported that welding fumes generated during shipbuilding and ship repair operations contain various metal elements, including cobalt (Co), cadmium (Cd), chromium (Cr), copper (Cu), iron (Fe), manganese (Mn), nickel (Ni), lead (Pb), zinc (Zn), molybdenum (Mo), vanadium (V), and antimony (Sb) [11]. Among them, Cr and Ni are confirmed human carcinogens, significantly increasing the risk of lung cancer in welders [12,13]; iron oxides cause pulmonary siderosis [1,14]; zinc is the primary causative agent of metal fume fever [15] (p. 2); and manganese can cause irreversible central nervous system disorders [16,17].

China’s position as the world’s largest shipbuilder [18] corresponds to a significant and extensively distributed welder workforce, deployed in numerous shipyards and related industries. Welding serves as the core process in shipbuilding, an inherently labor-intensive industry [19]. Welding fumes represent a primary occupational hazard, posing a severe threat to workers’ health [20], with risk being particularly prominent in shipbuilding. Confined spaces (such as ship compartments and final assembly sections) often suffer from inadequate ventilation, where the non-compliance rates for welding fume concentrations can reach 100% [21]. Globally, research on welding fume exposure has evolved from simple concentration monitoring to comprehensive characterization of particle size, chemical speciation, and health risk assessment. Driscoll [22] conducted a survey in Australian welding workplaces to establish baseline data of fume exposure concentrations. Takahashi [23] systematically measured fume particle size distributions and generation rates during the arc welding of cast iron, providing critical estimates for respirable dust formation. Brand [24] investigated the number size distribution of fine and ultrafine particles generated by diverse welding processes, highlighting the high prevalence of inhalable nanoparticles that pose unique inhalation risks. In China, domestic research has also begun to address these complexities. For instance, Yang Feng simulated welding operations in controlled experimental chambers to characterize the exposure features of gas shielded welding fumes. However, despite these isolated advances, a significant disparity remains when translating these findings to the actual shipbuilding industry. Current industry censuses and sentinel surveillance programs in China’s shipbuilding sector predominantly rely on a single indicator: total dust concentration [21,25]. Therefore, current research on welding fumes in the shipbuilding sector fails to provide a comprehensive, multi-dimensional risk assessment framework that simultaneously integrates dust concentration, particle size distribution, and detailed metal compositional analysis.

To bridge this gap, this study systematically characterized welding fume exposure in a representative Chinese shipyard through a comprehensive analysis of fume concentration, particle size distribution, and hazardous metals. The objective was to elucidate the distribution characteristics of welding fume hazards, quantify key exposure levels, and analyze the primary influencing factors. Ultimately, this study could provide data support and a theoretical basis for conducting precise occupational exposure risk assessments, evidence-based formulation of protective measures, safeguarding workers’ occupational health, and reducing the incidence of related occupational diseases.

Notably, the 2015 baseline data were initially collected to support the development of China’s “Diagnostic Criteria for Occupational Pulmonary Thesaurismosis Induced by Metal and Its Compound Dusts” (published in 2017) [26]. Following IARC’s 2017 classification of welding fumes as Group 1 carcinogens, we expanded our analytical approach beyond single-dimension reporting to adopt internationally recognized models for in-depth analysis of the full-dimensional matching data on “dust concentration–particle size distribution–metal composition”. The full-chain matched dataset from this study is scarce. The mainstream welding processes involved remain widely used today, and our findings can inform current prevention and control strategies, filling a gap in the field with both academic and public health significance.

## 2. Materials and Methods

### 2.1. Study Site and Survey Content

This study was conducted in 2015 at a large-scale shipbuilding enterprise. A systematic investigation was performed on welding fume exposure concentration, particle size distribution, and hazardous metal concentration. The enterprise encompassed several medium-sized shipyards and covered the three most prevalent welding processes in the shipbuilding industry: gas metal arc welding (GMAW), shielded metal arc welding (SMAW), and submerged arc welding (SAW) processes. It also featured diverse work sites such as indoor, outdoor, and confined spaces, making it highly representative of China’s shipbuilding sector.

This research was approved by the Ethics Committee of the National Institute of Occupational Health and Poison Control, the Chinese Center for Disease Control and Prevention, China (No. NIOHP201302). The approval was valid from January 2014 to December 2016, covering the entire study period. All participants signed the written informed consent prior to the study.

### 2.2. Dust Sampling and Measurement

Field sampling was performed in accordance with “Specifications of Air Sampling for Hazardous Substances Monitoring in the Workplace”(GBZ 159-2004) [27], with no fewer than two blank samples per batch. For area sampling, Gilian Gilair pumps (Sensidyne, St. Petersburg, FL, USA) were used at a constant flow rate of 5 L/min. Personal sampling was performed using AirChek 2000 portable pumps (SKC, Eighty Four, PA, USA) at a flow rate of 2 L/min. All pumps were calibrated before and after each sampling session using the Gilibrator 2 soap-film flow calibrator (Sensidyne, St. Petersburg, FL, USA). Throughout the sampling process, detailed records were maintained, including work locations, sampling types, welding processes, and sampling durations.

Dust sampling and laboratory analysis were conducted in accordance with “Determination of Dust in the Air of Workplace—Part 1: Total Dust Concentration” (GBZ/T 192.1-2007) [28], and “Determination of Dust in the Air of Workplace—Part 2: Respirable Dust Concentration” (GBZ/T 192.2-2007) [29]. Total dust sampling employed 30 mm sampling heads (Yilian, Suzhou, China), while respirable dust collection utilized pre-separators (SKC, USA) installed upstream the sampling heads with a cut-point of 7.07 µm aerodynamic diameter. Area sampling involved the collection of both total and respirable dust, whereas personal sampling targeted respirable dust only. Glass fiber filters were used for dust weight measurement, and mixed cellulose ester (MCE) filters were used for metal analysis. Formulas for calculating total dust and respirable dust concentrations are provided in Supplementary Material S1.

“Occupational Exposure Limits for Hazardous Agents in the Workplace—Part 1: Chemical Hazardous Agents” (GBZ 2.1-2019) [30] specifies the occupational exposure limit (OEL) for total particulate matter of welding fumes (termed “welding fumes” in the standard) as 4 mg/m^3^, which was applied to evaluate excessive welding fume levels in the workplace. It should be noted that the OEL for welding fumes remained unchanged at 4 mg/m^3^ in both GBZ 2.1-2007 [31] and GBZ 2.1-2019, ensuring methodological consistency and comparability of exposure assessments across the study timeline.

### 2.3. Arrangement of Sampling Points

To accommodate on-site workers’ shift arrangements, sampling was conducted at fixed welding workstations, where area and personal dust samples were collected continuously throughout the workday. Sampling points were established at workers’ regular operating positions, with at least one point established per production workshop. The sampling strategy encompassed: (1) different working sites—outdoor, indoor, and confined spaces, and (2) different welding processes, including the three most prevalent types in the shipyard—GMAW, SMAW, and SAW. For personal respirable dust sampling, workers from different work areas and welding processes were selected as subjects. Sampling pumps were consistently affixed to the upper chest to collect air samples from the workers’ breathing zone. To prevent membrane dislodgement from dust overload (>10 mg), the sampling heads were inspected hourly during personal sampling and replaced when significant dust accumulation was observed. Overall, 138 personal dust samples were collected from 97 sampling points. For multiple samples from the same sampling point, time-weighted averaging was applied to calculate both dust and metal concentrations at that point. The calculation formula is as follows:

(1) $$\mathrm{TWA}\;=\;\frac{\sum_{i}^{n}C_{i}\;T_{i}}{\sum T_{i}}$$

where TWA (mg/m^3^) is the time-weighted average concentration of dust or metal at that point, C_i_ (mg/m^3^) is the concentration in sample i, and T_i_ (h) is the sampling duration for sample i.

The details of the collected samples are summarized in Table 1. The average sampling duration of area sampling points was 3.45 h (range: 2.50–4.75 h), while personal sampling points averaged 3.75 h (range: 2.00–6.91 h).

### 2.4. Metals Analysis

We used Inductively Coupled Plasma–Optical Emission Spectrometry (ICP-OES) (5100 ICP-OES, Agilent, Santa Clara, CA, USA) and Inductively Coupled Plasma–Mass Spectrometry (ICP-MS) (NexIon 350D, PerkinElmer, Waltham, MA, USA) to quantify the concentrations of iron (Fe), manganese (Mn), zinc (Zn), nickel (Ni), copper (Cu), and chromium (Cr) in collected dust. Concentrations below the limit of detection (LOD) were replaced with LOD/2 for statistical analysis. The formula for calculating metal concentration was provided in Supplementary Materials S2.

### 2.5. Determination of Particle Size Distribution of Welding Fumes

The particle size distribution (0.25–32 μm) of welding fumes was measured using a portable aerosol optical particle size spectrometer (professional spectrometer 1.109, Grimm Aerosol Technik, Germany), which operates on light scattering principles for real-time aerosol monitoring. The sample flow rate was set at 1.2 L/min, with data recorded once every 60 s. Simultaneously, mass concentrations of particulate matter with aerodynamic diameters ≤ 1 μm (PM_1_), ≤2.5 μm (PM_2.5_), and ≤10 μm (PM_10_) were monitored in real-time throughout the sampling period. Welding fume particle size distribution was characterized across two working sites (outdoor, indoor), three distances from the welding spot (2 m, 10 m, 30 m), and three welding processes (GMAW, SMAW, SAW). The average sampling duration at each measurement point was approximately 1 h.

### 2.6. Health Risk Assessment

Based on the inhalation risk assessment methodology recommended by the United States Environmental Protection Agency (US EPA) [32], the exposure risks of six metals (Fe, Mn, Zn, Ni, Cu, Cr) in welding fumes in the shipyard were quantitatively evaluated.

The exposure concentration (EC) for exposures to the metals in air via the inhalation pathway was estimated using Equation (2):

(2) $$\mathrm{EC}\;=\;\frac{\mathrm{CA}\;\times \;\mathrm{ET}\;\times \;\mathrm{EF}\;\times \;\mathrm{ED}}{\mathrm{AT}}$$

where EC (mg/m^3^) is the chronic exposure concentration, CA (mg/m^3^) is the measured time-weighted average contaminant concentration in air, ET (h/day) is the exposure time, EF (days/year) is the exposure frequency, ED (years) is the exposure duration, and AT (lifetime in years × 365 days/year × 24 h/day) is the averaging time.

For non-cancer risk assessment, the hazard quotient (HQ) was calculated as follows:

(3) $$\mathrm{HQ}\;=\;\frac{\mathrm{EC}}{RfC}$$

where HQ (unitless) is the hazard quotient, EC (mg/m^3^) is the exposure concentration (see Equation (2)), and RfC (mg/m^3^) is the reference concentration. When HQ < 1, it indicates negligible non-cancer health effects. When HQ ≥ 1, it suggests a high likelihood of adverse non-carcinogenic health consequences.

For cancer risk assessment, the lifetime cancer risk (LCR) was calculated as follows:

LCR = IUR × EC (4)

where LCR (unitless) is the lifetime cancer risk, IUR (µg/m^3^)^−1^ is the inhalation unit risk, and EC (µg/m^3^) = exposure concentration (see Equation (2)). If LCR ≤ 10^−6^, it implies a negligible or absent cancer risk. If LCR > 10^−6^, it indicates a potential excess cancer risk, with higher values representing greater severity.

### 2.7. Statistical Analysis

Statistical analysis was conducted using SPSS 26.0 software (IBM Corp., Armonk, NY, USA). Prior to data analysis, normality was tested using the Shapiro–Wilk test (for sample sizes < 50) and the Kolmogorov–Smirnov test (for sample sizes ≥ 50). As most data exhibited non-normal distribution, they were described using the median (25th–75th percentiles). Group comparisons were conducted using the Kruskal–Wallis H test with Dunn’s post hoc test, Mann–Whitney U test for independent samples, and Wilcoxon Signed-Rank Test for paired samples. Spearman’s correlation analysis was used to assess the association between variables. Graphs were generated using R 4.2.2 (R Foundation for Statistical Computing, Vienna, Austria) and GraphPad Prism 10.6.0 (GraphPad Software, Inc., Boston, MA, USA). The test level α was set at 0.05 for all statistical analyses.

## 3. Results

### 3.1. Distribution of Welding Fume Concentration in the Shipyard

Welding fume concentration distribution in the shipyard is summarized in Table 2. Of 36 total dust samples, 17 samples exceeded the limit (4 mg/m^3^), with a non-compliance rate of 47.2%. For 17 non-compliance samples, the median concentration of these samples was 10.32 mg/m^3^ (range of 4.04–65.53 mg/m^3^).

Area sampling yielded median concentrations of 3.50 mg/m^3^ (total dust) and 3.22 mg/m^3^ (respirable dust), whereas personal respirable dust showed a median of 13.03 mg/m^3^. Significant differences in exposure levels were observed across welding sites (*p* < 0.05), with confined spaces exhibiting higher concentrations and a total dust non-compliance rate of 91.7% (11/12). The welding process also showed significant variations (*p* < 0.05), with GMAW demonstrating higher exposure concentrations and a total dust non-compliance rate of 54.8% (17/31).

### 3.2. Particle Size Distribution of Welding Fume in Shipyard Under Different Conditions

#### 3.2.1. Welding Sites

**Figure 1:**
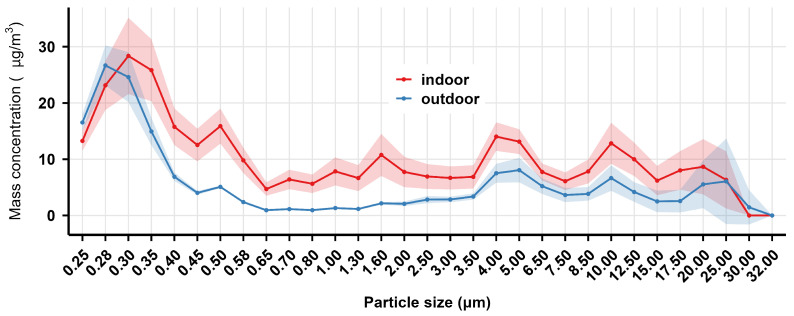
The mass concentration distribution of indoor and outdoor welding fume particles in the air at 2 m from the gas metal arc welding spot in the shipyard. The solid lines represent the mean mass concentration, while the shaded areas indicate the 95% confidence interval (CI), derived from optical particle size distribution.

**Figure 2:**
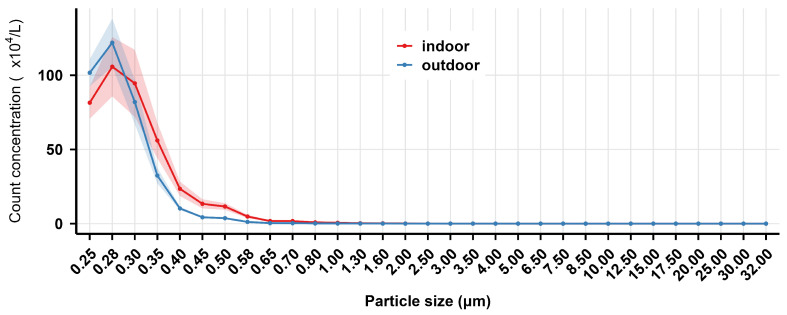
The count concentration distribution of indoor and outdoor welding fume particles in the air at 2 m from the gas metal arc welding spot in the shipyard. The solid lines represent the mean mass concentration, while the shaded areas indicate the 95% confidence interval (CI), derived from optical particle size distribution.

**Figure 3:**
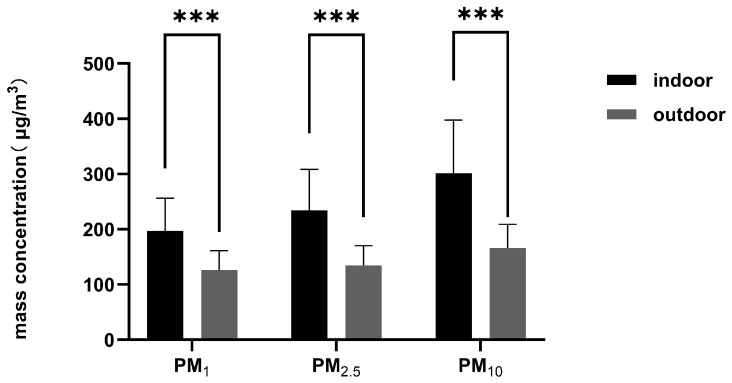
The particulate matter concentration of indoor and outdoor welding fume particles in the air at 2 m from the gas metal arc welding spot in shipyards, derived from optical particle size distribution. ***: *p* < 0.001.

The mass and count concentration distribution of indoor and outdoor welding fume particles (0.25–32.00 μm) in the air measured at 2 m from the GMAW spot in the shipyard are shown in Figure 1 and Figure 2, respectively. The results indicated that both the mass and count concentration of welding fumes were dominated by particles smaller than 1.00 μm in both indoor and outdoor sites. Within the <0.45 μm range, both mass and count concentration reached maximum values, with indoor concentrations generally exceeding outdoor levels. As particle size increased, mass concentrations showed an overall downward trend at both sites, while count concentrations declined sharply. As shown in Figure 3, the mass concentrations of indoor PM_1_, PM_2.5_, and PM_10_ were significantly higher than outdoor levels (*p* < 0.001).

In conclusion, the welding site affected the distribution of both mass concentration and count concentration of welding fumes, with the welding fumes at 2 m from the GMAW spot showing higher concentrations in indoor sites than in outdoor ones.

#### 3.2.2. Distances from the Welding Spot

**Figure 4:**
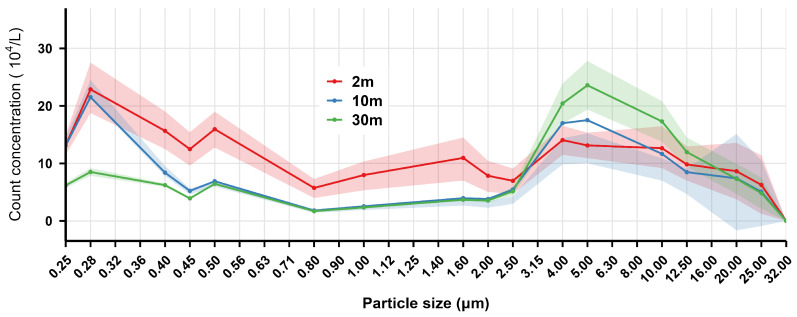
The mass concentration distribution of indoor welding fume particles in the air at different distances from the indoor gas metal arc welding spot in the shipyard. The solid lines represent the mean mass concentration, while the shaded areas indicate the 95% confidence interval (CI)), derived from optical particle size distribution.

**Figure 5:**
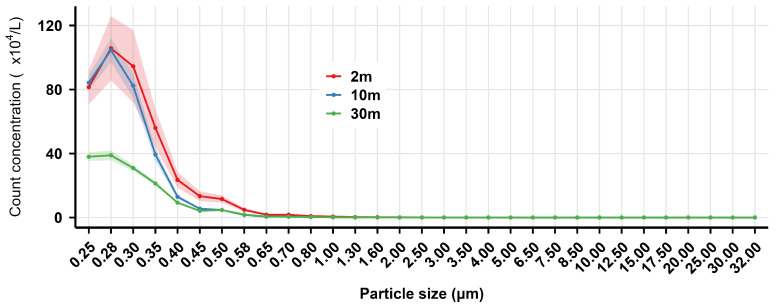
The count concentration distribution of indoor welding fume particles in the air at different distances from the indoor gas metal arc welding spot in the shipyard. The solid lines represent the mean mass concentration, while the shaded areas indicate the 95% confidence interval (CI), derived from optical particle size distribution.

The mass and count concentration distribution of welding fume particles (0.25–32.00 μm) in the air at different distances from the indoor GMAW spot in the shipyard are shown in Figure 4 and Figure 5, respectively.

As shown in Figure 4, the mass concentration reached its first peak in the 0.25–0.45 μm particle size range, then decreased significantly with increasing measurement distance (2 m > 10 m > 30 m). For particles sized 0.50–3.50 μm, the mass concentration trends at 10 m and 30 m were similar. A second peak occurred in the 4.00–8.50 μm range, where mass concentration at 30 m was higher than that at 10 m, which was higher than that at 2 m. The count concentration results (Figure 5) indicated that the particle size distribution at all three distances was dominated by particles smaller than 1.00 μm, with the highest peak occurring at 0.28 μm. The count concentrations decreased markedly with increasing measurement distance.

**Figure 6:**
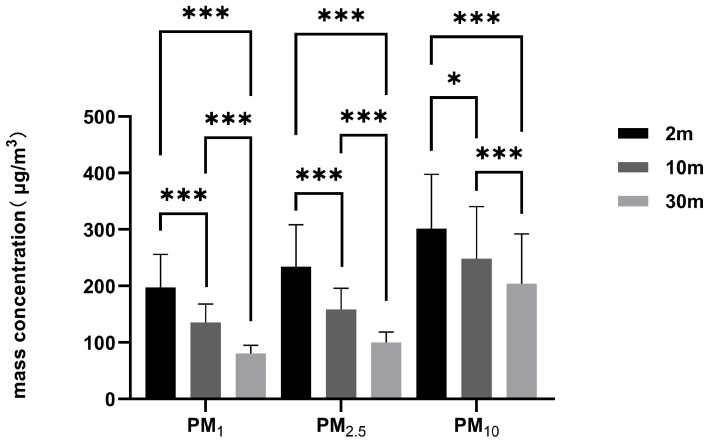
The particulate matter concentration distribution of indoor welding fume particles in the air at different distances from the indoor gas metal arc welding spot in the shipyard, derived from optical particle size distribution. *: *p* < 0.05; ***: *p* < 0.001.

Figure 6 demonstrates significant differences (*p* < 0.05) in PM_1_, PM_2.5_, and PM_10_ mass concentrations across all three distances. For all three particulate matter concentrations, the distribution pattern consistently followed 2 m > 10 m > 30 m.

In summary, distance was a key determinant of welding fume concentration. Both particle count and the mass concentrations of PM_1_, PM_2.5_, and PM_10_ demonstrated a consistent decline as distance increased.

#### 3.2.3. Welding Processes

**Figure 7:**
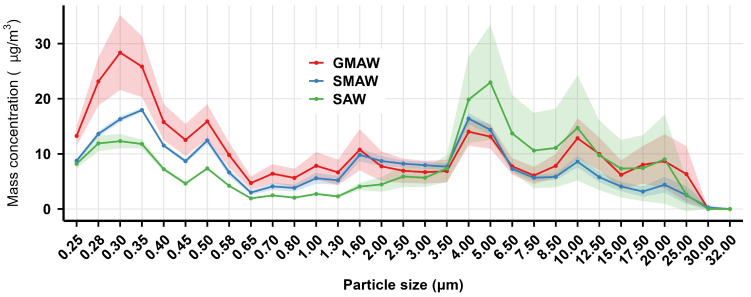
The mass concentration distribution of indoor welding fume particles in the air at 2 m from different welding processes in a shipyard. The solid lines represent the mean mass concentration, while the shaded areas indicate the 95% confidence interval (CI), derived from optical particle size distribution. GMAW is gas metal arc welding, SMAW is shielded metal arc welding, SAW is submerged arc welding.

**Figure 8:**
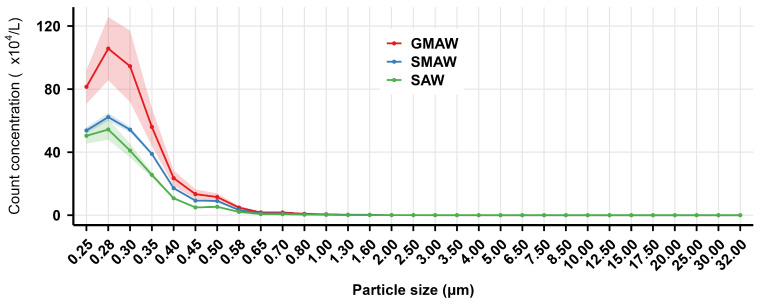
The count concentration distribution of indoor welding fume particles in the air at 2 m from different welding processes in a shipyard. The solid lines represent the mean mass concentration, while the shaded areas indicate the 95% confidence interval (CI), derived from optical particle size distribution. GMAW is gas metal arc welding, SMAW is shielded metal arc welding, SAW is submerged arc welding.

The mass and count concentration distribution of indoor welding fume particles (0.25–32.00 μm) in the air at 2 m from different welding processes in the shipyard are presented in Figure 7 and Figure 8, respectively.

As illustrated in Figure 7, the mass concentration from the three processes fluctuated with particle size. Within the 0.25–1.00 μm range, GMAW exhibited higher mass concentrations than SMAW and SAW. For particles larger than 5.00 μm, mass concentration differences between processes gradually diminished.

Figure 8 indicates that the particle size distribution from all three processes was dominated by particles smaller than 1.00 μm, with peak count concentration occurring at 0.28 μm. GMAW exhibited significantly higher count concentration for particles smaller than 1.00 μm compared to SMAW and SAW. As particle size increased, the count concentration decreased rapidly.

**Figure 9:**
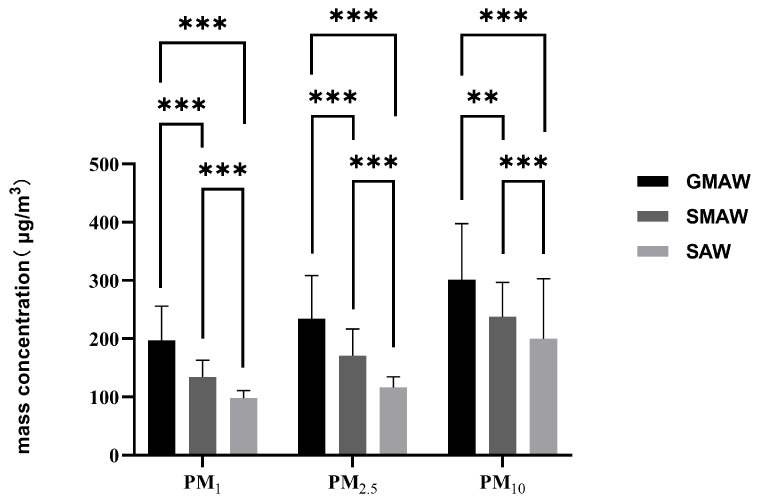
The particulate matter concentration distribution of indoor welding fume particles in the air at 2 m from different welding processes in the shipyard, derived from optical particle size distribution. GMAW is gas metal arc welding, SMAW is shielded metal arc welding, SAW is submerged arc welding. **: *p* < 0.01; ***: *p* < 0.001.

Figure 9 shows that the mass concentrations of PM_1_, PM_2.5_, and PM_10_ generated by the three welding processes exhibited significant differences (*p* < 0.01). For all three particulate matter concentrations, the distribution pattern consistently followed GMAW > SMAW > SAW.

Thus, welding processes significantly influenced both mass and count concentration distribution of welding fumes across particle sizes, with GMAW producing higher concentrations than SMAW and SAW.

#### 3.2.4. Interim Summary

Combined analysis of Figure 1 and Figure 4, and 7 revealed that welding fume mass concentration in the shipyard exhibited a typical bimodal distribution pattern: the first mass concentration peak occurred within the 0.25–0.45 μm particle size range, while the second peak appeared in the 4.00–6.50 μm range. With increasing particle size, the mass concentration fluctuated and decreased, approaching zero at 32 μm.

The results in Figure 2 and Figure 5, and 8 indicated that the count concentrations were highly concentrated in the <1.00 μm size range, decreasing rapidly with increasing particle size within the 0.25–1.00 μm range.

### 3.3. Concentrations of Various Airborne Metals at the Shipyard Welding Workplace

Samples were collected separately for area sampling (total dust and respirable dust) and personal sampling (respirable dust only). Table 3 presents the detection results of various metal concentrations in welding fumes in the shipyard. The metal concentration levels in area samples showed significant variations: Fe, Mn, and Zn concentrations were at the 10^2^ μg/m^3^ level, while Ni, Cu, and Cr concentrations were at the 10^0^ to 10^−1^ μg/m^3^ level. The concentrations of Fe, Mn, Zn, and Cu in personal samples were generally higher than those in area samples. Given that the total dust and respirable dust samples collected via area sampling were paired, the Wilcoxon Signed-Rank Test was employed. The results indicated that, in area samples, the concentrations of all six metals were significantly higher in respirable dust compared to total dust.

### 3.4. Correlation Analysis of Airborne Metals in the Shipyard Welding Workplaces

**Figure 10:**
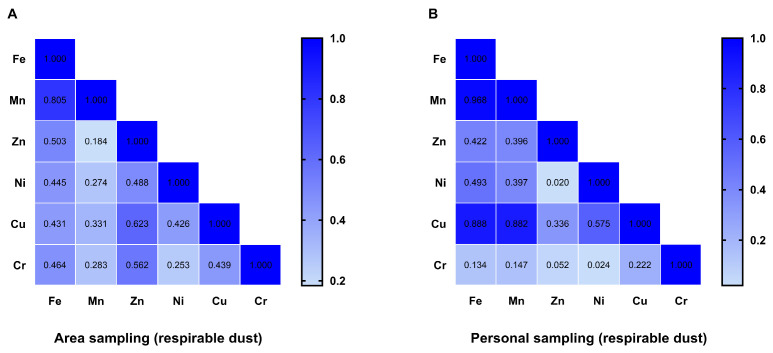
Spearman correlation analysis between various metals in the air of welding workplaces in a shipyard. (**A**) respirable dust from area sampling; (**B**) respirable dust from personal sampling.

Spearman correlation analysis was performed to assess the associations between various metals in the air of welding workplaces; the results are shown in Figure 10. For respirable dust from area sampling, the correlation between Fe and Mn was strong (*r* = 0.805, *p* < 0.01), while the correlations between Fe and Zn, Ni, Cu, and Cr were moderate. The correlations between Zn and Cu, as well as Cr, were moderate. The lowest correlation was observed between Mn and Zn (*r* = 0.184, *p* = 0.304).

For respirable dust from personal sampling, the correlation between Fe and Mn was further enhanced (*r* = 0.968, *p* < 0.01). The correlations of Cu with Fe and Mn were higher than those in respirable dust from area sampling. In contrast, the correlation between Cr and other metals was significantly reduced compared to respirable dust from area sampling.

### 3.5. Correlation Analysis Between Metal Concentrations and Workplace Conditions

#### 3.5.1. Welding Site

**Figure 11:**
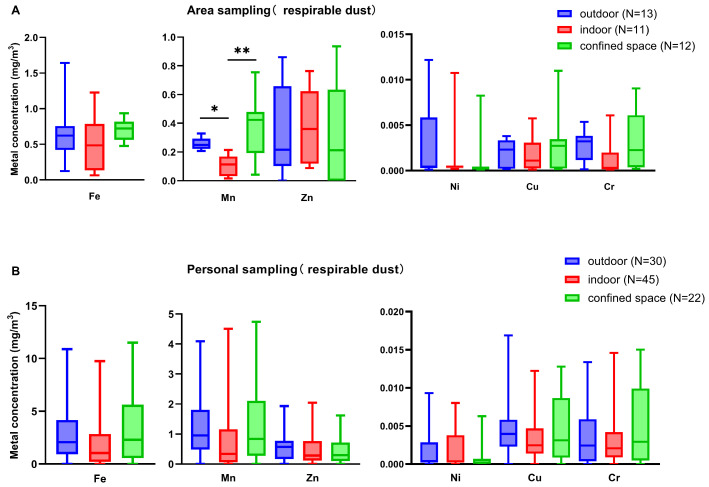
The metal concentration distribution of welding fumes in different welding sites in shipyard. Inter-group comparison excluding outliers, outliers < P_25_ − 1.5 IQR or outliers > P_75_ + 1.5 IQR; (**A**) respirable dust from area sampling; (**B**) respirable dust from personal sampling. *: *p* < 0.05; **: *p* < 0.01.

Figure 11 shows the metal concentration distribution of welding fumes at different welding sites in the shipyard. Metal concentrations measured via personal sampling were generally higher than those from area sampling. Confined spaces were identified as high-exposure zones for Fe and Mn. However, although personal exposure levels in confined spaces were numerically higher, the differences in metal concentrations across different welding sites did not reach statistical significance (*p* > 0.05).

#### 3.5.2. Welding Process

**Figure 12:**
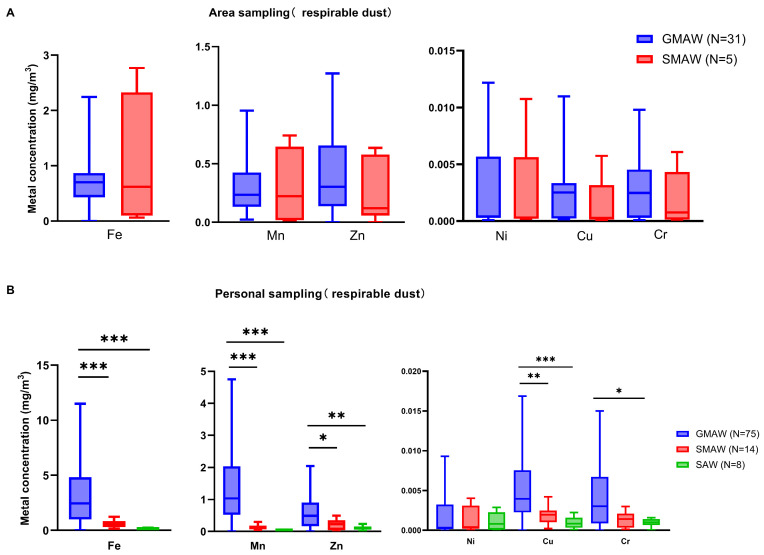
The metal concentration distribution of fume particles from different welding processes in shipyard. Inter-group comparison excluding outliers, outliers < P_25_ − 1.5 IQR or outliers > P_75_ + 1.5 IQR; (**A**) respirable dust from area sampling; (**B**) respirable dust from personal sampling. GMAW is gas metal arc welding, SMAW is shielded metal arc welding, SAW is submerged arc welding. *: *p* < 0.05, **: *p* < 0.01, ***: *p* < 0.001.

Figure 12 shows the metal concentration distribution of fume particles from different welding processes in the shipyard. For area sampling, there was no significant difference in metal concentrations between GMAW and SMAW. For personal sampling, metal concentrations varied across different welding processes, with the overall trend showing GMAW > SMAW > SAW.

### 3.6. Correlation Analysis of Dust Concentration and Metal Concentration Across Different Sampling Methods in the Shipyard

**Figure 13:**
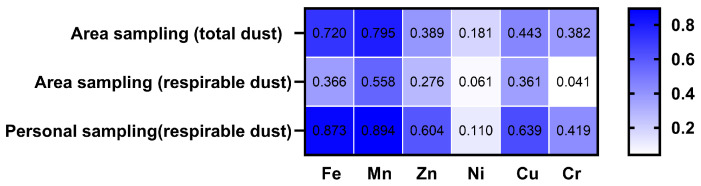
The Spearman correlation analysis of dust concentration and metal concentration across different sampling methods in the shipyard.

Figure 13 presents the Spearman correlation analysis results between dust concentration and metal concentration in dust across different sampling methods in the shipyard.

Both Fe and Mn showed strong correlations with all three dust types, whereas Ni exhibited extremely weak correlations with all dust types. Overall, the correlation between respirable dust from personal sampling and metal elements was slightly higher than that for area sampling respirable dust and total dust. However, Ni exhibited the strongest correlation with total dust from area sampling.

### 3.7. Inhalation Risk Assessment

The US EPA inhalation risk assessment was conducted following the methodology described in Section 2.6. The exposure assessment parameters included: CA from field measurements, ET (8 h/day) [33], EF (270 days/year) [34], ED (10 years, mean service length of shipyard welders from previous studies [35]), and AT (ED × 365 × 24 h for non-carcinogens; 70 × 365 × 24 h for carcinogens) [33], based on US EPA guidelines and previous studies. Detailed definitions and sources of all parameters are provided in Supplementary Material S3. Non-cancer risk was estimated for all metals, whereas cancer risk was calculated with Ni. Since only the total Cr content was measured, the Cr in this study was calculated as Cr (III).

#### 3.7.1. Non-Cancer Risk Assessment

Inhalation non-cancer risk assessment for exposure to metals at different working sites and across different welding processes is shown in Table 4.

Personal sampling generally yielded higher hazard quotient (HQ) values compared to area sampling. Mn was identified as the highest-risk element, with HQ values far exceeding 1. Ni and Cr were also classified as high-risk metals, with HQ > 1 observed at most sampling points. These results indicate a substantial risk of adverse non-carcinogenic health effects due to chronic exposure to Mn, Ni, and Cr. In contrast, the HQs of Fe, Zn, and Cu were all less than 1, indicating no non-cancer health concerns at current exposure levels.

**Figure 14:**
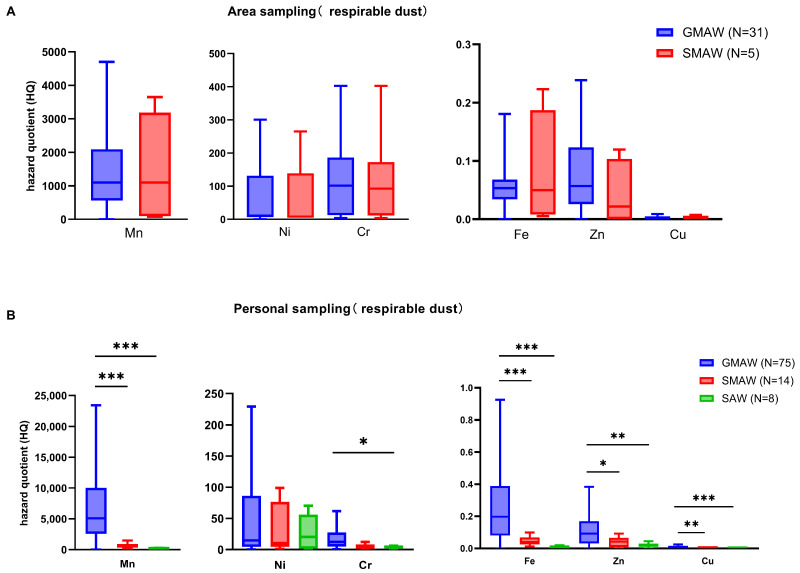
The hazard quotient (HQ) values for inhalation exposure to metals in different welding processes. Inter-group comparison excluding outliers, outliers < P_25_ − 1.5I QR or outliers > P_75_ + 1.5 IQR; (**A**) respirable dust from area sampling; (**B**) respirable dust from personal sampling. GMAW is gas metal arc welding, SMAW is shielded metal arc welding, SAW is submerged arc welding. *: *p* < 0.05, **: *p* < 0.01, ***: *p* < 0.001.

Inhalation non-cancer risk assessment for exposure to metals in different welding processes is shown in Figure 14. For different working processes, the HQs of metal elements generally followed the trend GMAW > SMAW > SAW.

#### 3.7.2. Cancer Risk Assessment

Inhalation cancer risk assessment for Ni across different welding processes is shown in Table 5. Both the ECs and LCRs derived from area sampling were higher than those from personal sampling. For different working processes, GMAW showed the highest median EC and LCR levels. Notably, the LCR values for all area sampling points exceeded the 10^−6^ carcinogenic risk threshold, indicating a potential carcinogenic risk. In contrast, the majority of personal sampling results remained below the 10^−6^ threshold.

## 4. Discussion

Welding fume concentration distribution is influenced by multiple complex factors. Shipyard operations are characterized by multi-process welding dispersion, high operational mobility that cannot be covered by fixed ventilation systems, and work in confined spaces such as ship hull compartments with poor ventilation. These characteristics hinder fume dispersion and facilitate the accumulation of fume pollutants. Based on the analysis results of welding fume concentration, particle size distribution, and metal composition, this study proposes the following systematic prevention and control recommendations according to the full-chain logic of “source control–operational control–personal protection–health monitoring–standard improvement.”

**Source Control**

Source control is primarily achieved by enhancing welding equipment, processes, and materials to reduce fume generation and mitigate health risks. Strategies should prioritize highly automated equipment to minimize worker exposure duration. Furthermore, substituting high-toxicity electrodes with non-toxic or low-toxicity alternatives is critical, especially considering that stainless steel fumes are significantly more toxic than low-carbon steel fumes [38]. Process selection also plays a vital role; for instance, Flux-Cored Arc Welding (FCAW) is known to create substantial fume emissions compared to other welding processes [39], necessitating stricter controls or alternative methods where possible.

**Operational Control**

The primary measures for operational control focus on deploying adequate ventilation facilities to expel welding fumes generated during operations, thereby minimizing the accumulation of welding fumes.

In the study, the concentration distribution of welding fumes revealed that confined spaces in the shipyard exhibited the highest fume concentrations, consistent with the research results of An Yu [40]. The main reason is the poor ventilation in confined spaces, which facilitates fume accumulation.

Regarding different welding processes, gas metal arc welding (GMAW) yields the highest welding fume concentrations. Furthermore, the results on particle size distribution and metal concentration demonstrated that welding processes exert a synergistic effect on both the particle size distribution and metal enrichment in welding fumes—manifesting as GMAW > SMAW > SAW across both dimensions. Mehrifar also found that GMAW resulted in higher metal concentrations, and welders involved in GMAW exhibited poorer pulmonary function compared to those involved in SMAW [41]. GMAW, characterized by concentrated electric arcs and high-temperature vaporization efficiency, generates significantly more fine particulate matter compared to SMAW and SAW. These small particles, due to their large specific surface area and strong surface activity, more readily adsorb or accumulate toxic metal components such as manganese, chromium, and iron volatilized during welding. The consistent impact of welding techniques on fume physicochemical properties (particle size distribution and metal composition) provides critical evidence for occupational health risk classification and the development of differentiated protective measures across various welding scenarios.

Therefore, confined spaces and GMAW are identified as key control targets in shipyards. Ventilation schemes should be optimized to reduce the accumulation of welding fumes—for example, by installing mobile fume extraction hoods [42]. In addition, technologies including ion charge local dust suppression and localized gas hoods should be adopted to further minimize fume accumulation [42].

The results of the particle size distribution of welding fume in the shipyard showed that the welding site affected the particle size distribution of welding fume. Indoor workplaces have limited space, making it easier for particles of various sizes to accumulate within short distances. In contrast, outdoor workplaces benefit from natural ventilation, which facilitates particle dispersion. As a result, the mass concentration and count concentration distributions of welding fumes were higher at indoor sites than at outdoor sites measured 2 m from the welding spots. Indoor workplaces should enhance ventilation by installing local purification systems and general ventilation systems to prevent dust accumulation. Outdoor workplaces should leverage natural ventilation advantages by strategically planning welding positions. During periods of low natural wind speed, portable exhaust equipment should be used to supplement ventilation.

**Personal Protection**

The particle size distribution analysis revealed that both the mass concentration and count concentration of welding fumes exhibited a pattern dominated by particles smaller than 1.00 μm, consistent with Wang Yilan’s opinions [2]. These particles can penetrate alveoli and cross the alveolar-capillary barrier into the circulatory system, causing lung damage. Long-term PM_1_ exposure increases the prevalence of asthma and pneumonia [43], while also elevating mortality risks in patients with chronic obstructive pulmonary disease (COPD), chronic bronchitis, and emphysema [44]. Personal protection should be implemented to minimize PM_1_ exposure among welders. Welders must strictly wear appropriate respirators of specific protection levels (such as N95, KN95, or higher-grade masks) or powered air-purifying respirators (PAPRs) during operations, and promptly change and clean work clothing after tasks to prevent contaminant transfer outside the work area. However, research has shown that some welders fail to comply with personal protective equipment protocols during actual production [22,45]. Therefore, it is essential to enhance the training of welders and raise their awareness of protective measures.

**Health Monitoring**

The concentration of respirable dust from personal sampling was higher than that of total dust from area sampling, consistent with findings from Huang Yunbiao [21] and An Yu [40]. At the metal element level, personal samples also showed higher exposure levels than area samples. This discrepancy may arise from the fact that welding operations are typically mobile, while area sampling pumps remain stationary, and personal sampling pumps move with welders during welding. Dust from area sampling reflect the overall welding fume pollution level in the workplace, whereas dust from personal sampling reflect the actual exposure levels of welders during operations. The Spearman correlation analysis of dust concentration and metal concentration under different sampling methods showed that the overall correlation of personal dust samples was slightly higher than that of area dust samples. This further indicated that personal exposure monitoring is more targeted than area monitoring in the occupational health risk assessment of welding fumes. However, although there were differences in metal concentrations between area samples and personal samples, the risk assessment results for area samples and personal samples were largely consistent.

Therefore, in occupational health surveys and risk assessments, area sampling can be adopted when constrained by conditions, but personal sampling enables a more accurate risk assessment. However, for determining actual exposure levels, especially in mobile welding operations, personal sampling can more precisely reflect workers’ true exposure to welding fumes.

This study only measured total Cr without determining Cr (VI) levels. Using Cr (III) for risk assessment might underestimate the health risks of Cr in the shipyard. Even when assuming the entirety of the measured total chromium to be the less toxic Cr (III), the HQs of Cr in area samples were all greater than 1, and most of those in personal samples were greater than 1, suggesting the potential for adverse health consequences due to Cr exposure. Future research should not only measure chromium content but also clarify chromium speciation to achieve a more precise risk assessment. The non-cancer risks of Mn and Ni were extremely high, suggesting that the exposure levels of Mn and Ni among shipyard welders far exceeded the safety thresholds corresponding to reference concentrations. Mn is an essential element, but excess exposure induces neurotoxic effects. It has been hypothesized that Mn-containing welding fumes are a possible neurological hazard. After inhalation, the absorbed Mn is transported in the blood and crosses the blood–brain barrier, and preferentially damages different areas of the brain [46]. Symptoms such as headache, sleep disturbance, and mood disorder can be observed after exposure to Mn. High-dose and chronic exposure to Mn can lead to the chronic neurological condition known as “manganism,” which is a neurological syndrome that resembles Parkinson’s disease [47]. Occupational health records should be established for workers, and annual specialized examinations for the nervous and respiratory systems should be conducted. Those showing early abnormal symptoms should be promptly reassigned from their positions and intervened in. The non-cancer risks of Fe, Zn, and Cu were found to be extremely negligible, a finding consistent with Lin Zhang’s study on welding fumes in electronic manufacturing workshops [48]. While specific targeted mitigation measures for these metals are not currently warranted, routine monitoring should still be emphasized to avoid overlooking abnormal increases in exposure levels caused by process adjustments and ventilation failures.

**Standard Improvement**

Analysis of metal concentrations in the shipyard welding fumes showed that respirable dust contained higher metal levels than total dust, indicating a preferential enrichment of metallic particles in the respirable fraction. This finding underscored the correlation between smaller particle sizes and elevated metal contamination. Consequently, these pollutants are more readily inhaled, posing heightened health risks associated with respirable dust.

China has established clear mandatory occupational exposure limits for the time-weighted average permissible concentration (PC-TWA) of total dust from area sampling of welding fumes in the “Occupational Exposure Limits to Hazardous Factors in the Workplace—Part 1: Chemical Hazardous Factors” (GBZ 2.1-2019). However, no corresponding specific limit standards have been established for respirable dust, which poses greater health risks. This results in limitations of the existing standard system in precisely controlling occupational health risks associated with welding operations. In contrast, the German Permanent Senate Commission for the Investigation of Health Hazards of Chemical Compounds in the Work Area (MAK Commission) has implemented graded precision control based on particle size distribution characteristics of welding fumes and toxicological research evidence [49]: it specifies a PC-TWA of 4 mg/m^3^ for inhalable dust and 0.3 mg/m^3^ for respirable dust in welding fumes. Respirable dust has smaller particle sizes and larger specific surface areas, making it more prone to accumulating toxic metal components such as Mn and Cr. Precise control of exposure levels is key to reducing occupational health risks in welding operations. Therefore, China urgently needs to draw on the standardized experience of developed countries such as Germany, accelerate the formulation and implementation of the PC-TWA limit for respirable dust in welding fumes, improve the occupational exposure limit system for welding fumes, achieve a shift from “total dust control” to “graded control”, further enhance the scientific rigor and targeted nature of occupational health protection, and effectively reduce the health exposure risks for workers.

It is important to note that this study focused on a single shipyard. Constraints related to sample representativeness and scope limit the generalizability of the findings to the entire shipbuilding industry. Future research should expand the sampling scale and diversity of worksites to validate and extend these conclusions, thereby providing a more robust scientific basis for the prevention and control of welding fume hazards. Moreover, ultrafine particles (<0.1 μm) were not assessed; future studies will target this fraction to clarify welding fume exposure profiles.

## 5. Conclusions

This study, conducted within a representative Chinese shipyard, systematically assessed welding fume exposure by examining particulate concentration (both mass and count), particle size distribution, and associated metal content. Results indicated significantly higher exposure concentrations in confined spaces and gas metal arc welding. Moreover, area sampling indicated that metal concentrations were higher in the respirable dust fraction than in total dust. An inhalation risk assessment of the metals revealed significant non-carcinogenic hazards from specific elements (e.g., Mn, Ni), while carcinogenic risks were also evaluated. Based on these results, targeted control strategies for welding fumes are proposed, highlighting the urgent need for China to establish specific occupational exposure limits for respirable welding fumes, offering a theoretical foundation for precise risk assessment and the development of effective protective measures.

## Figures and Tables

**Table 1:** Collection status and qualification rate of dust samples.

Sampling Type	Dust Type	Total Samples Collected (N)	Qualified Samples (N)	Qualification Rate (%)
Area sampling	Total dust	37	36	97.3
	Respirable dust	37	36	97.3
Personal sampling	Respirable dust	149	138	92.6
Total	-	223	210	94.2

Note: -: not applicable. N denotes the sample size; unqualified samples refer to those with unchanged or reduced filter membrane weight after sampling due to technical issues.

**Table 2:** Distribution of welding fume concentration in shipyard (mg/m^3^).

Classification	Area Sampling (Total Dust)			Area Sampling (Respirable Dust)		Personal Sampling (Respirable Dust)	
	N	Non-Compliance (%)	M (P_25_, P_75_)	N	M (P_25_, P_75_)	N	M (P_25_, P_75_)
welding site							
outdoor	13	4 (30.8)	2.65 (2.05, 8.02)	13	1.89 (1.55, 7.28)	30	15.55 (7.46, 25.02)
indoor	11	2 (18.2)	1.95 (0.48, 3.07)	11	2.40 (0.79, 3.31)	45	6.75(1.54, 22.38)
confined spaces	12	11 (91.7)	10.64 (4.46, 39.93) ^ab^	12	9.09 (3.26, 29.27) ^ab^	22	21.66 (8.35, 62.84) ^b^
welding process							
GMAW	31	17 (54.8)	4.08 (2.23, 10.32)	31	3.26 (1.89, 8.75)	75	16.92 (10.59, 32.18)
SMAW	5	0	1.95 (0.40, 3.16) ^c^	5	0.83 (0.65, 3.20) ^c^	14	2.08 (1.28, 3.25) ^c^
SAW	-	-	-	-	-	8	0.41 (0.36, 0.48) ^c^
summation	36	17 (47.2)	3.50 (1.95, 9.62)	36	3.22 (1.77, 8.30)	97	13.03 (3.47, 26.69)

Notes: -: not applicable. N denotes number of sampling points; M: median; P_25_: 25th percentile; P_75_: 75th percentile. GMAW is gas metal arc welding, SMAW is shielded metal arc welding, SAW is submerged arc welding. ^a^: compared with outdoor, *p* < 0.05; ^b^: compared with indoor, *p* < 0.05; ^c^: compared with GMAW, *p* < 0.05.

**Table 3:** Metal concentration in the air of welding workplaces in shipyard (μg/m^3^).

Metal	Area Sampling (Total Dust) N = 36	Area Sampling (Respirable Dust) N = 36	Personal Sampling (Respirable Dust) N = 97	*p*
Fe	447.01 (238.67, 840.12)	661.30 (427.78, 884.78)	1560.01 (539.40, 3598.35)	0.003 *
Mn	160.47 (99.89, 616.61)	222.44 (127.69, 424.05)	628.44 (151.64, 1598.73)	0.021 *
Zn	139.13 (0.29, 309.78)	300.31 (119.04, 628.85)	370.20 (120.90, 745.03)	<0.001 *
Ni	0.16 (0.13, 0.17)	0.40 (0.31, 5.30)	0.35 (0.16, 2.85)	<0.001 *
Cu	0.17 (0.09, 1.42)	2.13 (0.22, 3.08)	3.08 (1.66, 5.41)	0.007 *
Cr	0.17 (0.10, 2.29)	2.25 (0.30, 3.87)	2.30 (0.80, 4.99)	<0.001 *

Notes: N denotes the number of sampling points. Metal concentrations are expressed as the median (25th–75th percentiles); *p*: value of Wilcoxon Signed-Rank Test about total dust and respirable dust from area sampling; *: *p* < 0.05.

**Table 4:** Inhalation non-cancer risk assessment for exposure to metals [median (range)].

		Area Sampling (Respirable Dust)		Personal Sampling (Respirable Dust)	
	RfC (mg/m^3^)	EC (mg/m^3^)	HQ	EC (mg/m^3^)	HQ
Fe	3.0625 ^a^	0.163 (<0.001~0.683)	0.053 (<0.001~0.223)	0.385 (<0.001~2.833)	0.126 (<0.001~0.925)
Mn	0.00005 ^b^	0.055 (0.004~0.235)	1096.976 (80.438~4698.172)	0.155 (<0.001~1.170)	3099.159 (<0.001~23,400.768)
Zn	1.312 ^a^	0.074 (<0.001~0.313)	0.056 (<0.001~0.239)	0.091 (<0.001~0.504)	0.070 (<0.001~0.384)
Ni	0.00001 ^b^	0.098 (0.023~3.004) × 10^−3^	9.814 (<0.001~300.374)	0.087(<0.001~2.295) × 10^−3^	11.457 (<0.001~229.48)
Cu	0.162 ^a^	0.525 (0.027~1.415) × 10^−3^	0.003(<0.001~0.009)	0.760 (<0.001~4.158) × 10^−3^	0.005 (<0.001~0.026)
Cr	0.00006 ^b^	0.555 (0.024~2.416) × 10^−3^	92.488 (4.009~402.614)	0.568 (<0.001~3.701) × 10^−3^	9.467 (<0.001~61.679)

Note: RfC is reference concentration, EC is exposure concentration, HQ is hazard quotient. ^a^: data from reference [36]; ^b^: data from reference [37].

**Table 5:** Inhalation cancer risk assessment for Ni in different welding processes [median (range)].

		Area Sampling (Respirable Dust)		Personal Sampling (Respirable Dust)	
	IUR (μg/m^3^)	EC (μg/m^3^)	LCR	EC (μg/m^3^)	LCR
GMAW	0.00026	0.015 (0.003~0.429)	3.925 (0.862~27.510) × 10^−6^	0.003 (<0.001~0.081)	0.677 (0.020~21.017) × 10^−6^
SMAW	0.00026	0.011 (0.007~0.379)	2.963 (1.818~98.420) × 10^−6^	0.003 (<0.001~0.035)	0.621 (0.118~9.060) × 10^−6^
SAW	0.00026	-	-	0.001 (<0.001~0.025)	0.321 (0.077~6.463) × 10^−6^
total	0.00026	0.016 (0.003~0.429)	4.066 (0.862~111.568) × 10^−6^	0.004 (<0.001~0.081)	0.654 (0.020~21.017) × 10^−6^

Note: -: not applicable. IUR is the inhalation unit risk, EC is exposure concentration, LCR is the lifetime cancer risk; GMAW is gas metal arc welding, SMAW is shielded metal arc welding, SAW is submerged arc welding.

## Data Availability

The data presented in this study are available on request from the corresponding author.

## Supplementary Materials

The following supporting information can be downloaded at: https://www.mdpi.com/article/10.3390/toxics14030259/s1, S1: Calculation of dust concentration; S2: Calculation of metal concentration; Table S1: microwave digestion conditions for environmental sample pretreatment; Table S2: detection limits of metal elements in dust samples; S3: Essential parameters associated with health risk assessment; Table S3: definition, typical values, and source of information [34,35,37].

## Abbreviations

The following abbreviations are used in this manuscript:

COPD	chronic obstructive pulmonary disease
NIOSH	National Institute for Occupational Safety and Health
Co	cobalt
Cd	cadmium
Cr	chromium
Cu	copper
Fe	iron
Mn	manganese
Ni	nickel
Pb	lead
Zn	zinc
Mo	molybdenum
V	vanadium
Sb	antimony
GMAW	gas metal arc welding
SMAW	shielded metal arc welding
SAW	submerged arc welding
ICP-OES	Inductively Coupled Plasma–Optical Emission Spectrometry
ICP-MS	Inductively Coupled Plasma–Mass Spectrometry
PM_1_	particulate matter with aerodynamic diameters ≤ 1 μm
PM_2.5_	particulate matter with aerodynamic diameters ≤ 2.5 μm
PM_10_	particulate matter with aerodynamic diameters ≤ 10 μm
US EPA	United States Environmental Protection Agency
EC	exposure concentration
CA	contaminant concentration
ET	exposure time
EF	exposure frequency
ED	exposure duration
AT	averaging time
HQ	hazard quotient
RfC	reference concentration.
IUR	inhalation unit risk
PC-TWA	time-weighted average permissible concentration

## References

[1] Riccelli M.G., Goldoni M., Poli D., Mozzoni P., Cavallo D., Corradi M. (2020). Welding Fumes, a Risk Factor for Lung Diseases. Int. J. Environ. Res. Public Health.

[2] Wang Y.L., Gang B.Q. (1994). Modern Occupational Health.

[3] Saadiani E., Sadeghi-Yarandi M., Nasiri A., Kalantary S. (2025). Assessing Long-Term Impacts of Occupational Welding Fume Exposure on Respiratory Health: A 5-Year Retrospective Cohort Analysis. J. Occup. Environ. Med..

[4] Galarneau J., Beach J., Cherry N. (2025). Respiratory III-Health and Welding Exposures: A Canadian Cohort Study. Am. J. Ind. Med..

[5] Tung N.T., Lai C.-H., Pan C.-H., Chen W.-L., Wang C.-C., Liang C.-W., Chien C.-Y., Chuang K.-J., Thao H.N.X., Dung H.B. (2022). Associations of PM2.5 with Chronic Obstructive Pulmonary Disease in Shipyard Workers: A Cohort Study. Aerosol Air Qual. Res..

[6] Momen N.C., Baker M., Driscoll T., Li J., Martínez-Silveira M.S., Turner M.C., Viegas S., Villeneuve P.J., Pega F. (2025). The effect of occupational exposure to welding fumes on trachea, bronchus, and lung cancer: A supplementary analysis of regular occupational exposure and of occasional occupational exposure based on the systematic review and meta-analysis from the WHO/ILO Joint estimates of the work-related burden of disease and Injury. Environ. Int..

[7] Tsuji M., Koriyama C., Ishihara Y., Isse T., Ishizuka T., Hasegawa W., Goto M., Tanaka R., Kakiuchi N., Hori H. (2023). Associations between welding fume exposure and neurological function in Japanese male welders and non-welders. J. Occup. Health.

[8] Wang C.G., Zhao X.Q., Jiao H., Li G.L., Xu Z.W., Sun Z.G., Yang W., Cheng J.Y. (2018). Analysis of cardiovascular system damage in welding workers. China Occup. Med..

[9] IARC Working Group on Humans (2018). Welding, Molybdenum Trioxide, and Indium Tin Oxide.

[10] Cao Z., He L., Luo Y., Tong X., Zhao J., Huang K., Chen Q., Jiao L., Liu Y., Geldsetzer P. (2025). Burden of chronic respiratory diseases and their attributable risk factors in 204 countries and territories, 1990–2021: Results from the global burden of disease study 2021. Chin. Med. J. Pulm. Crit. Care Med..

[11] National Institute for Occupational Safety and Health (NIOSH) (2023). Evaluation of Exposure to Metals and Noise During Shipbuilding and Ship Repair Operations: Report No. 2023-0084-3425 [R/OL]. https://www.cdc.gov/niosh/hhe/reports/pdfs/2023-0084-3425.pdf.

[12] Pesch B., Kendzia B., Pohlabeln H., Ahrens W., Wichmann H.E., Siemiatycki J., Taeger D., Zschiesche W., Behrens T., Jöckel K.H. (2019). Exposure to Welding Fumes, Hexavalent Chromium, or Nickel and Risk of Lung Cancer. Am. J. Epidemiol..

[13] Yatera K., Morimoto Y., Ueno S., Noguchi S., Kawaguchi T., Tanaka F., Suzuki H., Higashi T. (2018). Cancer Risks of Hexavalent Chromium in the Respiratory Tract. J. UOEH.

[14] Song P.P., Zhang H., Sun X.W., Luo L.M., Zhang J.J., Han J.J. (2025). Six cases of pulmonary siderosis caused by iron and its compounds. Zhonghua Lao Dong Wei Sheng Zhi Ye Bing Za Zhi.

[15] Brenner B.E., Keyes D. (2025). Metal Fume Fever.

[16] Sidoryk-Wegrzynowicz M., Aschner M. (2013). Manganese toxicity in the central nervous system: The glutamine/glutamate-γ-aminobutyric acid cycle. J. Intern. Med..

[17] O’Neal S.L., Zheng W. (2015). Manganese Toxicity Upon Overexposure: A Decade in Review. Curr. Environ. Health Rep..

[18] Xie Y., Yin Q., Jin W.C. (2021). Review and Future Prospects of China’s Shipbuilding Industry During the “13th Five-Year Plan” Period. World Shipp..

[19] Yang W.T. (2012). Identification and Analysis of Occupational Disease Hazard Factors in Shipyards. J. Saf. Sci. Technol..

[20] An Y., Song Y., He Z.L. (2015). Occupational Health Problems and Protective Measures in the Shipbuilding Industry. Occup. Health.

[21] Huang Y.B., Wang Y., Shi Y. (2011). Analysis of Welding Fume Detection Results in the Shipbuilding Industry. J. Environ. Occup. Med..

[22] Driscoll T.R., Paine S., Fritschi L., Nguyen H., Carey R.N. (2025). Occupational exposure to welding fume in Australian workplaces. Ind. Health.

[23] Takahashi J., Nakashima H., Fujii N. (2020). Fume particle size distribution and fume generation rate during arc welding of cast iron. Ind. Health.

[24] Brand P., Lenz K., Reisgen U., Kraus T. (2013). Number size distribution of fine and ultrafine fume particles from various welding processes. Ann. Occup. Hyg..

[25] Jiang S., Jiang Y.Q., Yu Y.T., Sun Z.X., Sheng F.S., Wang Y.M. (2019). Exposure Level and Influencing Factors of Welding Fumes in Sentinel Enterprises in Songjiang District, Shanghai, 2014–2018. J. Environ. Occup. Med..

[26] (2017). Diagnosis of Occupational Pulmonary Thesaurosis Induced by Dust of Metal and Its Compounds (Tin, Iron, Antimony, Barium and Its Compounds).

[27] (2004). Specifications of Air Sampling for Hazardous Substances Monitoring in the Workplace.

[28] (2007). Method for Determination of Dust in the Air of Workplace—Part 1: Total Dust Concentration.

[29] (2007). Method for Determination of Dust in the Air of Workplace—Part 2: Respirable Dust Concentration.

[30] (2019). Occupational Exposure Limits for Hazardous Agents in the Workplace—Part 1: Chemical Hazardous Agents.

[31] (2007). Occupational Exposure Limits for Hazardous Agents in the Workplace—Part 1: Chemical Hazardous Agents.

[32] (2009). US EPA Risk Assessment Guidance for Superfund, Volume I: Human Health Evaluation Manual (Part f, Supplemental Guidance for Inhalation Risk Assessment).

[33] U.S. EPA Exposure Factors Handbook 2011 Edition (Final Report). U.S. Environmental Protection Agency, Washington, DC, EPA/600/R-09/052F, 2011. https://cfpub.epa.gov/ncea/risk/recordisplay.cfm?deid=236252.

[34] Dhiman R., Prakash A., Saroj S., Sahoo P., Ambekar A., Kore S.D., Thajudeen T., Guttikunda S.K. (2026). Occupational health risks from welding emissions: Exposure and deposition of PM_10_, PM_2.5_, and ultrafine particles across welding methods. Environ. Sci. Adv..

[35] Li Y., Wang H.Q., Zhang M.B., Ni C.H. (2024). Association between exposure to multiple metals and lung function in welders by multi-pollu-tant statistical models. Int. J. Occup. Environ. Med..

[36] Dehghani F., Omidi F., Fallahzadeh R.A., Pourhassan B. (2021). Health risk assessment of occupational exposure to heavy metals in a steel casting unit of a steelmaking plant using Monte-Carlo simulation technique. Toxicol. Ind. Health.

[37] US EPA Regional Screening Levels (RSLs)—Generic Tables. https://www.epa.gov/risk/regional-screening-levels-rsls-generic-tables.

[38] Leonard S.S., Chen B.T., Stone S.G., Schwegler-Berry D., Kenyon A.J., Frazer D., Antonini J.M. (2010). Comparison of stainless and mild steel welding fumes in generation of reactive oxygen species. Part. Fibre Toxicol..

[39] Quecke E., Quemerais B., Hashisho Z. (2023). Review of welding fume emission factor development. Ann. Work Expo. Health.

[40] An Y., Zhang Y., Wang Y.Y., Song X.Y., Tian H.F., He Z.L., Du Y., Shao X.C., Wang Y. (2018). Monitoring results of welding fumes of a shipbuilding enterprise in Dalian City. Occup. Health.

[41] Mehrifar Y., Zamanian Z., Pirami H. (2019). Respiratory Exposure to Toxic Gases and Metal Fumes Produced by Welding Processes and Pulmonary Function Tests. Int. J. Occup. Environ. Med..

[42] Huang J.W., Shen A.L., Zhang Z.S. (2015). Hazards of occupational exposure to welding fume and related control measures. Occup. Health Emerg. Rescue.

[43] Lu W., Sun H., Xu R., Wei J., Shi C., Zhong C., Liu Y., Zhou Y. (2025). Ambient PM(1) on COPD mortality: Insights from a population-based study. J. Epidemiol. Community Health.

[44] Adiministrator Y., Wu M., Li Y., Liu X. (2022). Influence of PM_1_ exposure on total and cause-specific respiratory diseases: A systematic review and meta-analysis. Environ. Sci. Pollut. Res..

[45] Koirala B., Rijal B., Kc S., Nepal S., Khadka A., Karki A., Joshi S., Basnet S., Adhikari U., Neupane R. (2025). Occupational health risks and safety awareness among welders in Nepal, a qualitative study. BMJ Open.

[46] Alici N., Nadir Öziş T., Çeliker G., Birlik Aktürk T. (2022). Welder’s lung and brain MRI findings in manganese-exposed welders. Med. Lav..

[47] Racette B.A., McGee-Minnich L., Moerlein S.M., Mink J.W., Videen T.O., Perlmutter J.S. (2001). Welding-related parkinsonism: Clinical features, treatment, and pathophysiology. Neurology.

[48] Zhang L., Yu J.M., Shan X.Y., Shao J., Ye H.P. (2023). Characterization of welding fume and airborne heavy metals in electronic manufacturing workshops in Hangzhou, China: Implication for occupational population exposure. Environ. Sci. Pollut. Res..

[49] Gao F., An S.K. (2021). Comparative analysis of harmful chemical factors for welding fume standards of occupational exposure limits(OELs)domestic and overseas. Electr. Weld. Mach..

