# Supplementary material for: Welding Fumes in a Chinese Shipyard: Exposure Characteristics and Occupational Health Risk Assessment
Source: Toxics. 2026 Mar 16;14(3):259. doi: 10.3390/toxics14030259 (PMC13030628; doi:10.3390/toxics14030259)
Supplement: Supplementary file 1 [file toxics-14-00259-s001.zip › toxics-4150100-supplementary.pdf]

**Supplementary Materials**

S1: *Calculation of dust concentration*

Calculate the concentration of welding fumes using the equation from S1:

$$C=\frac{m_2-m_1}{V\times t}\times 1000 \qquad (S1)$$

where C (mg/$m^3$) is the concentration of harmful substances in the air, $m_2$ (mg) is the weight of the filter membrane after sampling, $m_1$ (mg) is the weight of the filter membrane before sampling, V (L/min ) is the sampling flow rate, and t (L/min ) is the sampling time.

S2: *Calculation of metal concentration*

Place the sampled microporous membrane into the digestion vessel of the microwave digestion system, sequentially add 2.5 mL of nitric acid and 1 mL of hydrogen peroxide, then seal the vessel for digestion under the conditions specified in Table 1. After the digestion vessel has cooled, transfer the digestion solution quantitatively to a stoppered graduated tube and dilute to the 25.00 mL mark before detection.

Calculate the metal content in welding fumes using the equation from S2:

$$C=\frac{25C_0}{V_0} \qquad (S2)$$

where C (mg/$m^3$) is the concentration of metals and their compounds in the air, 25 (ml) is the volume of the sample solution, $C_0$ (µg/ml) is the measured concentration of the metal in the sample solution, and $V_0$ (L) is the standard sampling volume.

**Table S1.** Microwave digestion conditions for environmental sample pretreatment

| step | digestion power/W | heating-up time /min | digestion temperature / °C |
|---|---|---|---|
| 1 | 1000 | 0-15 | room temperature-180 |
| 2 | 1000 | 15-25 | 18 |

**Table S2.** Detection limits of metal elements in dust samples (µg/mL)

| metal | Limit of detection |
|---|---|
| Fe | 0.003 |
| Mn | 0.003 |
| Zn | 0.003 |
| Ni | 0.007 |
| Cu | 0.004 |
| Cr | 0.005 |

 https://doi.org/10.3390/toxics14030259

S3: *Essential parameters associated with health risk assessment*

**Table S3.** Definition, typical values, and source of information

| parameter | definition and units | value | source |
|---|---|---|---|
| CA | contaminant concentration in air (mg/m$^3$) | measured metal concentration in shipyard | field investigation and laboratory testing |
| ET | exposure time (hours/day) | 8 | US EPA [1] |
| EF | exposure frequency (days/year) | 270 | [2] |
| ED | exposure duration (years) | 10, mean length of service of welders in shipyard | previous studies [3] |
| AT | averaging time (h) | non-carcinogens AT = ED × 365 days/year × 24 hours/day<br>carcinogens AT = 70 year × 365 days/year × 24 hours/day | US EPA [1] |

1. U.S. EPA. Exposure Factors Handbook 2011 Edition (Final Report). U.S. Environmental Protection Agency, Washington, DC, EPA/600/R-09/052F, 2011.https://cfpub.epa.gov/ncea/risk/recordisplay.cfm?deid=236252

2. Dhiman, R.; Prakash, A.; Saroj, S.; Sahoo, P.; Ambekar, A.; Kore, S.D.; Thajudeen, T.; Guttikunda, S.K. Occupational health risks from welding emissions: exposure and deposition of $PM_{10}$, $PM_{2.5}$, and ultrafine particles across welding methods. *Environ. Sci.* Adv. 2026, 5, 59–77. https://doi.org/10.1039/d5va00142k.

3. Li, Y.; Wang, H.Q.; Zhang, M.B.; Ni, C.H. Association between exposure to multiple metals and lung function in welders by multi-pollutant statistical models. *Int J Occup Environ Med* 2024, 41(3), 251-258, doi:10.11836/JEOM23322.
